# A role for two‐component signaling elements in the Arabidopsis growth recovery response to ethylene

**DOI:** 10.1002/pld3.58

**Published:** 2018-05-09

**Authors:** Brad M. Binder, Hyo Jung Kim, Dennis E. Mathews, Claire E. Hutchison, Joseph J. Kieber, G. Eric Schaller

**Affiliations:** ^1^ Department of Biochemistry and Cellular & Molecular Biology University of Tennessee Knoxville Tennessee; ^2^ Department of Biological Sciences Dartmouth College Hanover New Hampshire; ^3^ Center for Plant Aging Research Institute for Basic Science (IBS) Daegu Korea; ^4^ Department of Molecular, Cellular, and Biomedical Sciences University of New Hampshire Durham New Hampshire; ^5^ Department of Biology University of North Carolina Chapel Hill North Carolina; ^6^ Present address: William Harvey Research Institute Queen Mary University of London Charterhouse Square London EC1M 6BQ UK

**Keywords:** Arabidopsis, ethylene, histidine kinase, phosphotransfer protein, receptor, response regulator

## Abstract

Previous studies indicate that the ability of Arabidopsis seedlings to recover normal growth following an ethylene treatment involves histidine kinase activity of the ethylene receptors. As histidine kinases can function as inputs for a two‐component signaling system, we examined loss‐of‐function mutants involving two‐component signaling elements. We find that mutants of phosphotransfer proteins and type‐B response regulators exhibit a defect in their ethylene growth recovery response similar to that found with the loss‐of‐function ethylene receptor mutant *etr1‐7*. The ability of two‐component signaling elements to regulate the growth recovery response to ethylene functions independently from their well‐characterized role in cytokinin signaling, based on the analysis of cytokinin receptor mutants as well as following chemical inhibition of cytokinin biosynthesis. Histidine kinase activity of the receptor ETR1 also facilitates growth recovery in the ethylene hypersensitive response, which is characterized by a transient decrease in growth rate when seedlings are treated continuously with a low dose of ethylene; however, this response was found to operate independently of the type‐B response regulators. These results indicate that histidine kinase activity of the ethylene receptor ETR1 performs two independent functions: (a) regulating the growth recovery to ethylene through a two‐component signaling system involving phosphotransfer proteins and type‐B response regulators and (b) regulating the hypersensitive response to ethylene in a type‐B response regulator independent manner.

## 1 INTRODUCTION

Two‐component signaling systems involve histidine kinases, response regulators, and sometimes histidine‐containing phosphotransfer proteins (Mizuno, 2005; Schaller, Shiu, & Armitage, 2011). Two‐component signaling systems are found in bacteria, archaea, fungi, slime molds, and plants (Mizuno, 2005; Schaller et al., 2011). The two‐component signaling system is so named because, in its simplest form, it incorporates a receptor histidine kinase and a response regulator (Gao & Stock, 2009; Stock, Robinson, & Goudreau, 2000). The receptor histidine kinase autophosphorylates on a conserved histidine residue in response to an environmental stimulus, and the phosphate is then transferred to a conserved aspartic acid residue within the receiver domain of a response regulator. Response regulators frequently serve as transcription factors, with phosphorylation modulating their ability to control gene expression. Plants make use of a permutation of the two‐component system known as the multistep phosphorelay (Schaller, Kieber, & Shiu, 2008). As found in plants, the multistep phosphorelay typically incorporates three components: (a) a hybrid receptor kinase that contains both histidine kinase and receiver domains in one protein, (b) a histidine‐containing phosphotransfer protein, and (c) a separate response regulator. In Arabidopsis, these are referred to as ARABIDOPSIS HISTIDINE KINASEs (AHKs), ARABIDOPSIS HISTIDINE‐CONTAINING PHOSPHOTRANSMITTERs (AHPs), and ARABIDOPSIS RESPONSE REGULATORs (ARRs). The two‐component signaling system has an established role in mediating cytokinin signal transduction in plants (Gruhn & Heyl, 2013; Hwang, Sheen, & Müller, 2012; Kieber & Schaller, 2014; To & Kieber, 2008; Werner & Schmülling, 2009), but a potential role in mediating ethylene signal transduction is still unclear despite the fact that several ethylene receptors have histidine kinase activity (Gamble, Coonfield, & Schaller, 1998; Moussatche & Klee, 2004).

The ethylene receptor family of plants is related to histidine kinases, containing sensor domains near their N‐termini and histidine kinase‐like domains in the C‐terminal halves. In Arabidopsis, the ethylene receptor family consists of five members that divide into two subfamilies based on phylogenetic analysis and some shared structural features. Subfamily 1 is composed of ETHYLENE RESPONSE1 (ETR1) and ETHYLENE RESPONSE SENSOR1 (ERS1), and subfamily 2 is composed of ETR2, ERS2, and ETHYLENE INSENSITIVE4 (EIN4) (Chang & Stadler, 2001; Chen, Etheridge, & Schaller, 2005; Schaller & Kieber, 2002). The subfamily‐1 receptors have canonical histidine kinase domains and exhibit histidine kinase activity based on in vitro analysis (Gamble et al., 1998; Moussatche & Klee, 2004), whereas the subfamily‐2 receptors contain diverged histidine kinase‐like domains and exhibit serine/threonine kinase activity based on in vitro analysis (Chen et al., 2009; Moussatche & Klee, 2004). Three of the five receptors are hybrid receptors (ETR1, ETR2, and EIN4) that contain receiver domains with all the conserved residues required for functioning in a multistep phosphorelay. In spite of these conserved features, no substantive role has been identified for the multistep phosphorelay in ethylene signaling (Binder, O'Malley, et al., 2004; Hall et al., 2012; Hass et al., 2004; Mason et al., 2005; Qu & Schaller, 2004). Instead, genetic analysis indicates that the primary elements functioning downstream of the ethylene receptors are the Raf‐like kinase CONSTITUTIVE TRIPLE RESPONSE1 (CTR1), the transmembrane protein EIN2, and the EIN3 family of transcription factors (Alonso, Hirayama, Roman, Nourizadeh, & Ecker, 1999; Chao et al., 1997; Huang, Li, Hutchison, Laskey, & Kieber, 2003; Ju et al., 2012; Kieber, Rothenberg, Roman, Feldman, & Ecker, 1993; Qiao et al., 2012; Solano, Stepanova, Chao, & Ecker, 1998). However, it has recently been shown that ETR1 and ETR2 also signal independently of CTR1, supporting the possibility for other, noncanonical signaling pathways (Bakshi et al., 2018).

If not involved in the primary signaling pathway, what role does the histidine kinase activity of the ethylene receptors play in signal transduction? Several studies suggest that the enzymatic activity of ETR1 modulates ethylene signal output (Binder, O'Malley, et al., 2004; Hall et al., 2012; Qu & Schaller, 2004; Street et al., 2015). This might seem to imply that a multistep phosphorelay operates downstream of the receptors; however, the histidine kinase and receiver domains of the receptors physically interact with CTR1 and EIN2 and autophosphorylation may also modulate interactions with the established ethylene signaling pathway (Binder, Chang, & Schaller, 2012; Bisson & Groth, 2010; Clark, Larsen, Wang, & Chang, 1998; Gao et al., 2003). It is thus necessary to specifically evaluate the role of two‐component signaling elements in ethylene‐mediated responses to resolve their level of contribution.

One situation in which histidine kinase activity of the ethylene receptors appears to play a modulating role is in the ability of seedlings to recover normal growth following cessation of ethylene treatment (Binder, O'Malley, et al., 2004). Treatment of wild‐type etiolated seedlings with ethylene inhibits their growth rate; upon removal of ethylene, the seedlings return to their basal growth rate within two hours. Loss‐of‐function receptor mutants for the family members with receiver domains (ETR1, ETR2, and EIN4) all result in a slower recovery to normal growth rate following removal of ethylene, the slow growth recovery phenotype being particularly strong in the *etr1‐7* mutant. In addition, the double mutant *etr1‐7;ers1‐2* is delayed in its ability to recover normal growth rate, and this can be rescued by introducing a wild‐type version of ETR1 but not by a kinase‐inactive version of ETR1 (Binder, O'Malley, et al., 2004).

These results suggest that histidine kinase activity and, by extension, the multistep phosphorelay may mediate the ability of seedlings to recover normal growth following cessation of ethylene treatment. We tested this hypothesis by performing a kinetic analysis of the ethylene growth response and recovery in mutants of Arabidopsis two‐component signaling elements. Results from this study indicate that histidine kinase activity of the ethylene receptor ETR1 performs two independent functions. First, it regulates the growth recovery to ethylene through a two‐component signaling system involving AHPs and type‐B ARRs. Second, it also plays a role in regulating growth recovery during a “hypersensitive” response to ethylene, which is characterized by a transient decrease in growth rate when seedlings are treated with a continuous very low dose of ethylene, but does so in a type‐B ARR‐independent manner. The potential mechanisms underlying these differences in histidine kinase‐mediated regulation are discussed.

## 2 MATERIALS AND METHODS

### 2.1 Plant materials

Loss‐of‐function mutants analyzed were the *etr1‐7* ethylene receptor mutant (Hua & Meyerowitz, 1998), *AHK* double mutants constructed from published T‐DNA insertion lines (Argueso et al., 2012), *AHP* mutants *ahp5‐2*,* ahp2 ahp3*, and *ahp2 ahp3 ahp5‐2* (Hutchison et al., 2006), type‐B *ARR* mutants *arr2‐4*,* arr10‐2 arr12‐1*, and *arr2‐2 arr10‐2 arr12‐1* (Mason et al., 2005), and are all of the Arabidopsis ecotype Col‐0. Construction of the transgenic *etr1‐7 ers1‐2* lines containing wild‐type *ETR1* (*gETR1*) or kinase‐inactivated ETR1 (*getr1‐HGG*) was as described (Wang, Hall, O'Malley, & Bleecker, 2003).

### 2.2 Kinetic analysis of hypocotyl growth rate

Measurements were performed using two‐day‐old etiolated Arabidopsis seedlings as described previously (Binder, O'Malley, et al., 2004). To examine the growth response and recovery kinetics to ethylene, 10 μl/L ethylene was introduced 1 hr after measurements were initiated and then removed 2 hr later. To examine the hypersensitive growth response to ethylene, a concentration of 8.7 nl/L ethylene was used. All data represent the mean of at least three seedlings for the recovery analysis and of at least four seedlings for the hypersensitive response analysis. The Kolmogorov–Smirnov test, performed with JMP 10.0, was used to assess the significance of differences in growth curves. For kinetic recovery analysis, the 3‐5 hr time points were compared for significance; for the hypersensitive response, the 1.5‐3.5 hr time points were compared for significance.

### 2.3 Hypocotyl growth response to cytokinin

Dose–response for the effect of the cytokinin *trans*‐zeatin on hypocotyl elongation of 4‐d‐old dark‐grown seedlings was performed as described (Argyros et al., 2008). Seedlings were grown on vertical plates on MS media with 1% (w/v) sucrose, along with the indicated concentrations of cytokinin, scanned, and lengths measured using ImageJ software. All data represent the mean of at least nine seedlings.

### 2.4 Accession numbers

Sequence data from this article can be found in the EMBL/GenBank data libraries under accession number(s): AHK2 (AT5G35750), AHK3 (AT1G27320), AHK4 (AT2G01830), AHP2 (AT3G29350), AHP3 (AT5G39340), AHP5 (AT1G03430), ARR2 (AT4G16110), ARR10 (AT4G31920), ARR12 (AT2G25180), ERS1 (AT2G40940), ETR1 (AT1G66340).

## 3 RESULTS

### 3.1 Two‐component signaling elements regulate the growth recovery to ethylene

**Figure 1:**
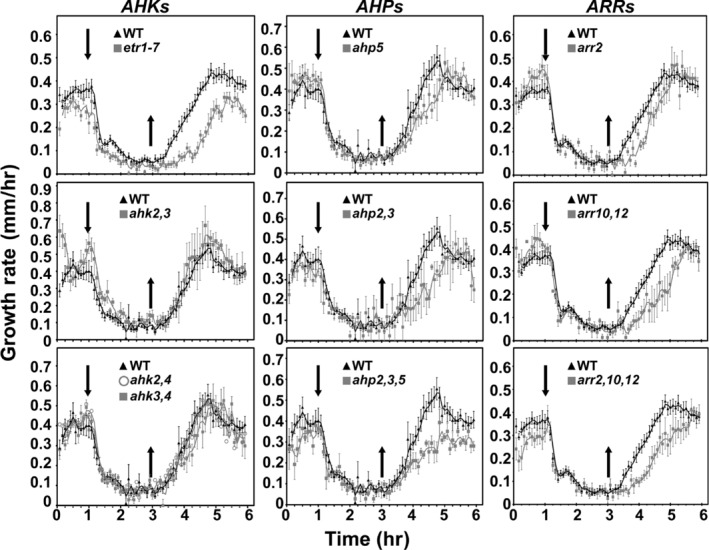
Growth kinetics of two‐day‐old etiolated Arabidopsis hypocotyls containing mutations in two‐component signaling elements. Short‐term growth kinetic analysis in response to 10 μl/L ethylene was performed on mutants involving receptors (column 1), AHPs (column 2), and type‐B ARRs (column 3) and compared to the wild type. Ethylene was introduced one hour after measurements were initiated (down arrow) and then removed two hours later (up arrow). For receptor mutants, we examined the *etr1‐7* mutant as well as *ahk* double mutants. For *ahp* mutants, we examined *ahp5‐2*,* ahp2 ahp3*, and *ahp2 ahp3 ahp5‐2*. For type‐B *arr* mutants, we examined *arr2‐4*,* arr10‐2 arr12‐1*, and *arr2‐2 arr10‐2 arr12‐1*. Error bars indicate *SE* (*n* ≥ 3)

We examined short‐term changes in seedling growth response and recovery to ethylene using two‐day‐old etiolated seedlings. Under these growth conditions, wild‐type seedlings exhibit a growth rate of approximately 0.4 mm/hr in the absence of ethylene (Figure 1). Treatment with 10 μl/L exogenous ethylene results in a rapid decrease in the growth rate, initiated within 15 min of the treatment and reaching a new steady‐state growth rate approximately 75 min after ethylene addition. As has been observed previously (Binder, Mortimore, Stepanova, Ecker, & Bleecker, 2004; Binder, O'Malley, et al., 2004), the growth response has two kinetic phases: a rapid inhibition response lasting for approximately 15 min, followed by a slower inhibition response that lasts for approximately 60 min. Removal of ethylene results in a recovery of seedling growth rate, with the initial growth rate being attained approximately 90 min after ethylene removal.

Loss‐of‐function mutations in the ethylene receptors *ETR1*,* ETR2*, or *EIN4* affect these growth recovery kinetics to ethylene (Binder, O'Malley, et al., 2004). As shown in Figure 1, the loss‐of‐function *etr1‐7* mutant exhibits a normal growth response to ethylene, but is significantly delayed in its recovery kinetics compared to wild type (*p* < 0.0001), taking approximately 30 min longer than wild type to attain its initial growth rate. We examined loss‐of‐function mutants involving the histidine kinase‐linked cytokinin receptors AHK2, AHK3, and AHK4 to determine whether the slow growth recovery phenotype was unique to ethylene receptor mutants (Figure 1). Because there is functional redundancy in the cytokinin receptor family, we examined the double mutants *ahk2 ahk3*,* ahk2 ahk4*, and *ahk3 ahk4*, all of which have demonstrated effects on shoot and root growth (Argueso et al., 2012; Higuchi et al., 2004; Nishimura et al., 2004; Riefler, Novak, Strnad, & Schmülling, 2006). All three *ahk* double mutant combinations exhibited ethylene growth response and recovery kinetics similar to those observed in wild type (Figure 1), indicating that ethylene receptors play a role in mediating these growth responses that does not require activity of the cytokinin receptors.

Arabidopsis contains five genes encoding phosphotransfer proteins (*AHP1*,* AHP2*,* AHP3*,* AHP4*, and *AHP5*) that are predicted to contain the conserved histidine for phosphorylation (Schaller et al., 2008). Single loss‐of‐function mutants do not display significant effects on cytokinin signaling, but higher order mutants involving *ahp1*,* ahp2*,* ahp3*, and *ahp5* result in decreased cytokinin sensitivity (Hutchison et al., 2006). We analyzed mutants involving *ahp2*,* ahp3*, and *ahp5* for their ethylene growth response and recovery kinetics (Figure 1). All the mutants exhibited an ethylene response similar to wild type, but the *ahp5*,* ahp2 ahp3*, and *ahp2 ahp3 ahp5* mutants exhibited a significantly slower growth recovery phenotype (*p* < 0.0001).

Arabidopsis contains 11 type‐B response regulators (type‐B ARRs), which serve as transcription factors to mediate the final step in the multistep phosphorelay (Schaller et al., 2008). *ARR1*,* ARR2*,* ARR10*, and *ARR12* are broadly expressed and based on genetic analysis contribute the most to cytokinin signal transduction (Argyros et al., 2008; Ishida, Yamashino, Yokoyama, & Mizuno, 2008; Mason, Li, Mathews, Kieber, & Schaller, 2004; Mason et al., 2005). *ARR2* has been implicated in ethylene signaling (Hass et al., 2004), and we therefore focused our analysis on *arr2* mutants (Figure 1). As with the other mutants examined, all the type‐B *arr* mutants exhibited an ethylene response similar to wild type, but the *arr2*,* arr10 arr12*, and *arr2 arr10 arr12* mutants exhibited a significantly slower growth recovery phenotype (*p* < 0.0001).

### 3.2 Two‐component signaling elements regulate ethylene growth recovery independently from their role in cytokinin signaling

**Figure 2:**
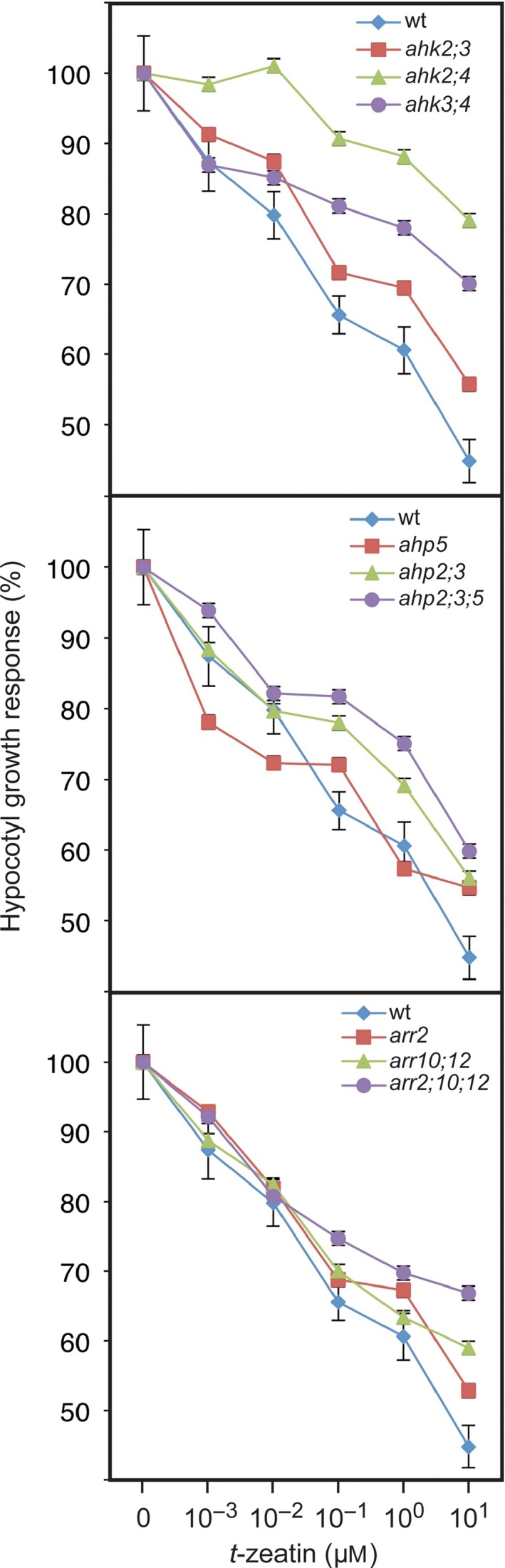
Hypocotyl growth response of two‐component mutants to cytokinin. Dose–response for the effect of the cytokinin *t*‐zeatin on hypocotyl elongation of four‐day‐old etiolated seedlings, performed as described (Argyros et al., 2008). The hypocotyl length of each line in the presence of cytokinin is expressed as a percentage of the control. Error bars indicate *SE* (*n* ≥ 9); error bars not shown if smaller than symbol

We performed several controls to confirm that the two‐component signaling elements regulate the growth recovery to ethylene independently from their role in cytokinin signaling. First, as described earlier, double mutants of the cytokinin receptors do not exhibit the same ethylene recovery phenotype as mutants involving the downstream AHPs and ARRs, which is not what would be predicted if cytokinin played a role in the ethylene response (Figure 1). We note that we could not test the *ahk* triple mutant because it is infertile and needs to be propagated in a segregating population, and the homozygous triple mutant cannot be differentiated phenotypically from other genotypes in the etiolated seedlings used for the kinetic analysis. To confirm the lack of correlation between the effects of the mutants on the cytokinin and ethylene responses, we specifically examined how mutations in the cytokinin receptors (*AHK*s), *AHP*s, and type‐B *ARR*s affect the hypocotyl growth response to cytokinin (Figure 2), doing so because our kinetic results are based on a hypocotyl growth response to ethylene. We observed that the *ahk* double mutants *ahk2 ahk4* and *ahk3 ahk4* were less responsive to cytokinin than any of the *ahp* and type‐B *arr* mutant combinations we tested, even though the *ahp* and *arr* mutants exhibit a stronger effect on ethylene growth recovery kinetics.

**Figure 3:**
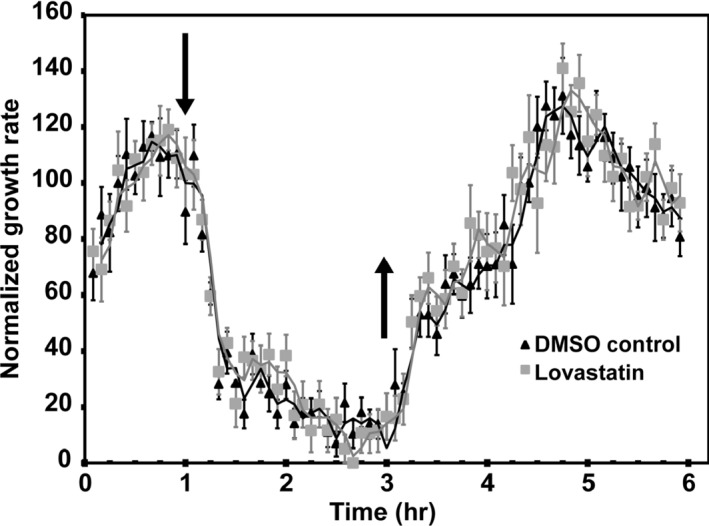
Lovastatin does not affect growth kinetics of two‐day‐old etiolated Arabidopsis hypocotyls. Short‐term growth kinetic analysis in response to 10 μl/L ethylene was performed on wild‐type seedlings grown in the absence (DMSO control) or the presence of 1 μM lovastatin. Data were normalized to growth rate in air prior to treatment with ethylene to facilitate the comparison. Unnormalized growth rates in air were 0.33 ± 0.01 mm/hr for the control and 0.24 ± 0.01 mm/hr for the lovastatin‐treated seedlings. Error bars indicate *SE* (*n* ≥ 3)

As an alternative approach to assess a potential role for cytokinin in the ethylene growth response, we performed kinetic analysis in the presence of the cytokinin biosynthesis inhibitor lovastatin (Crowell & Salaz, 1992). Lovastatin is an inhibitor of the cytosolic pathway for isoprenoid biosynthesis, one of the two pathways that generate the isopentenyl groups used in the biosynthesis of cytokinins. Treatment of four‐day‐old dark‐grown seedlings with 1 μM lovastatin inhibited hypocotyl growth, reducing hypocotyl length from 9.87 mm (±0.15 *SE*) for the untreated control to 7.10 mm (±0.07 *SE*) for the lovastatin‐treated seedlings (i.e., a 28% reduction in growth). When examined by kinetic analysis, the control seedlings exhibiting a growth rate of 0.33 mm/hr (± 0.01 *SE*) compared to 0.24 mm/hr (± 0.01 *SE*) for the lovastatin‐treated seedlings (i.e., a 23% reduction in growth rate). This lovastatin‐induced reduction in hypocotyl growth is consistent with that previously observed (Nagata, Suzuki, Yoshida, & Muranaka, 2002), is indicative of the efficacy of the inhibitor, and also still allowed sufficient growth to perform kinetic analysis (Figure 3). We found that the ethylene growth response and recovery kinetics are not affected by lovastatin, consistent with these growth responses being independent of cytokinin (Figure 3). Thus, the effects we observe for the mutants and lovastatin on the ethylene response do not correlate with their effects on cytokinin signaling responses.

### 3.3 Histidine kinase activity of ETR1 regulates the hypersensitive response to ethylene in a type‐B ARR‐independent manner

**Figure 4:**
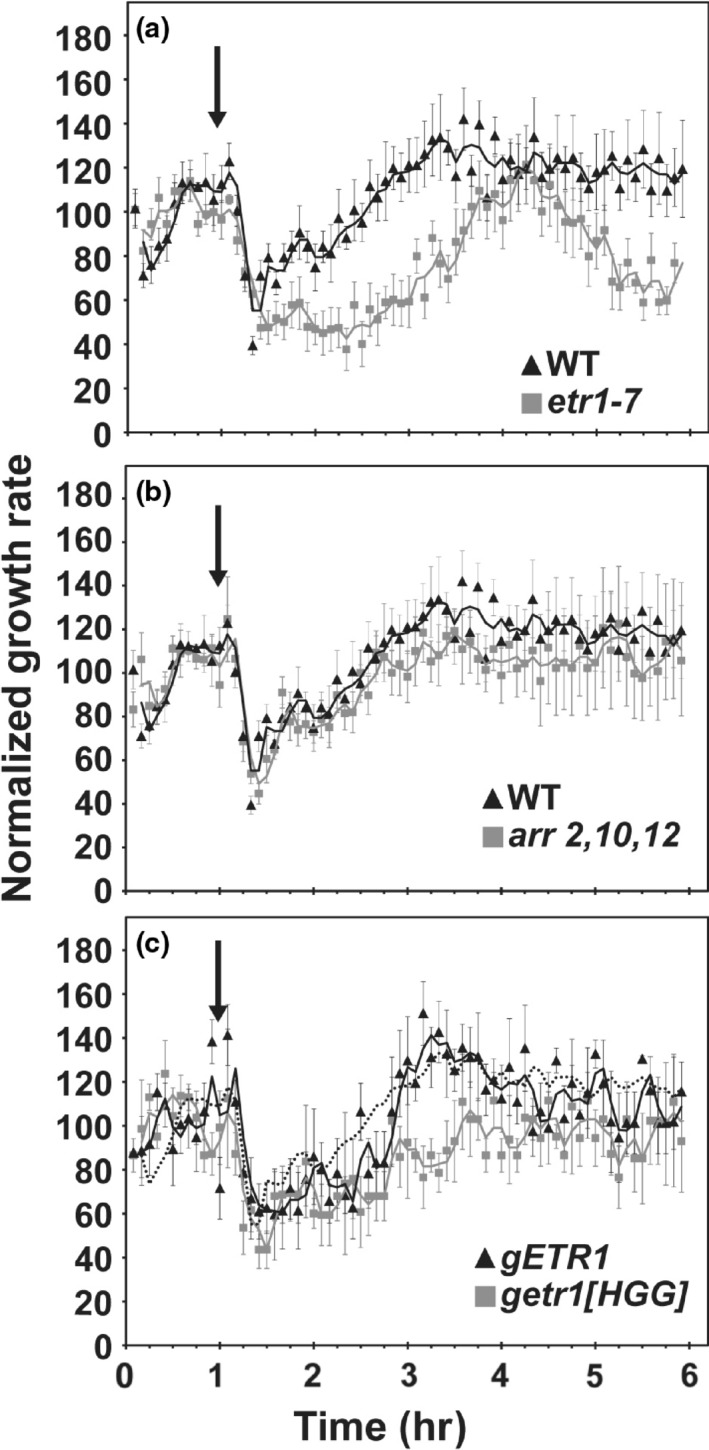
Kinetic analysis of the hypersensitive response to ethylene. Growth kinetic analysis was performed with 8.7 nl/L ethylene introduced 1 hr after measurements were initiated (down arrow). The hypersensitive response of *etr1‐7* (a), *arr2‐2 arr10‐2 arr12‐1* (b), and transgenic *etr1‐7 ers1‐2* lines containing wild‐type *ETR1* (*gETR1*), or kinase‐inactive ETR1 (*getr1‐HGG*) (c) are all compared to wild type. The dotted line in panel C shows data for wild type for comparison. Data were normalized to growth rate in air prior to treatment with ethylene to facilitate comparisons. Error bars indicate *SE* (*n* ≥ 4)

The first phase for ethylene growth inhibition, characterized by a rapid decrease in seedling growth, can be differentiated from the second slower response phase based on its hypersensitivity to ethylene (Binder, Mortimore, et al., 2004). As shown in Figure 4, wild‐type seedlings treated with 8.7 nl/L ethylene (an approximately 1000‐fold lower dose than that used in Figure 1) exhibit a rapid but transient decrease in their growth rate, recovering to their pretreatment growth rate in approximately 2 hr, even though the seedlings are still in the presence of ethylene. We examined the receptor mutant *etr1‐7* to determine whether ETR1 plays a role in the hypersensitive ethylene response. The *etr1‐7* mutant responded similarly to wild type, but its reduced growth rate was significantly prolonged compared to wild type (*p* < 0.0001), requiring approximately 1 hr longer to return to the initial growth rate (Figure 4). To determine whether type‐B ARR transcription factors might play a role in the hypersensitive response, we examined the ethylene response of *arr2 arr10 arr12* but observed similar growth kinetics to wild type (Figure 4). To determine whether the histidine kinase activity of ETR1 plays a role in the hypersensitive response, we made use of the subfamily‐1 loss‐of‐function mutant *etr1‐7 ers1‐2* transformed with wild‐type *ETR1* (*gETR1*) or a kinase‐inactive *ETR1* (*getr1‐HGG*) (Binder, O'Malley, et al., 2004). The *gETR1* line exhibited a hypersensitive response similar to wild type, but the *getr1‐HGG* line exhibited a significantly prolonged hypersensitive response (*p* < 0.0001), suggesting that histidine kinase activity plays a role in the response.

## 4 DISCUSSION

**Figure 5:**
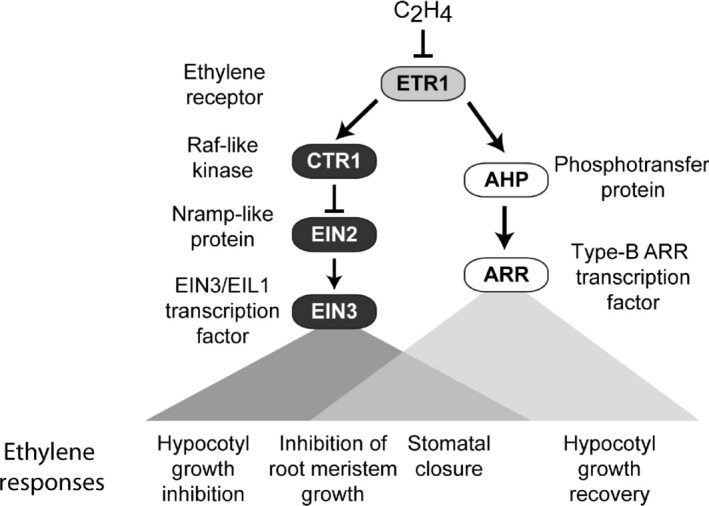
Model for ethylene signal transduction. Signal output from the ethylene receptors diverges to regulate the CTR1/EIN2/EIN3 and AHP/ARR pathways. ETR1 is shown here as a representative ethylene receptor that has histidine kinase activity. Ethylene responses may be dependent on an individual pathway or have varying contributions from both pathways

These results indicate that histidine kinase activity of the ethylene receptor ETR1 performs two independent functions: (a) regulating the growth recovery to ethylene through a two‐component signaling system involving AHPs and type‐B ARRs and (b) regulating the hypersensitive response to ethylene in a type‐B ARR‐independent manner. Several points can be made about the role of the two‐component system in ethylene signaling. First, loss‐of‐function mutants in ethylene receptors *ETR1*,* ETR2*, and *EIN4* (Binder, O'Malley, et al., 2004), the *AHP*s, and the type‐B *ARR*s, although having no effect on the initial ethylene response, all affect ethylene growth recovery kinetics in a similar manner, consistent with their functioning in the same regulatory pathway (Figure 5). The 45‐min delay in recovery time observed in some of the mutants is substantive when one considers that growth recovery usually occurs in less than 2 hr, and indicates that the two‐component signaling pathway plays a significant role in mediating the plant's responsiveness to changes in its phytohormone environment. Second, there is functional overlap within members of each two‐component family for mediating the ethylene recovery response, similar to what has been found in the cytokinin signaling pathway (Schaller et al., 2008; To & Kieber, 2008; Werner & Schmülling, 2009), a finding consistent with physical interactions being detected for multiple AHPs with ETR1 and the type‐B ARRs (Dortay et al., 2008; Scharein, Voet‐van‐Vormizeele, Harter, & Groth, 2008; Urao, Miyata, Yamaguchi‐Shinozaki, & Shinozaki, 2000). Third, given the role of type‐B ARRs as transcription factors, the effect of the two‐component signaling pathway is likely to be on transcriptional output from ethylene signaling, potentially regulating the expression of genes involved in the growth response (Hass et al., 2004). Fourth, the AHPs and type‐B ARRs that function in ethylene signaling also play roles in cytokinin signaling (Kieber & Schaller, 2014; To & Kieber, 2008; Werner & Schmülling, 2009), raising the possibility of cross talk between these phytohormone signaling pathways as well as the question as to whether and how specificity is obtained when utilizing the same signaling elements.

Our results complement prior studies that have implicated two‐component signaling elements in the regulation of ethylene responses, including effects on hypocotyl growth, stomatal closure, and root meristem development (Figure 5). The type‐B response regulator ARR2 was previously implicated in ethylene responses based on an *arr2* mutant exhibiting a hyposensitive ethylene response for the inhibition of hypocotyl elongation, and the ARR2 protein being phosphorylated in an ETR1‐dependent manner and binding to a promoter element for the ethylene‐induced gene ERF1 (Hass et al., 2004). In contrast to this study by Hass et al. (2004), we do not observe an effect of *arr2* mutants in the hypocotyl growth response assay, either as a long‐term effect (Mason et al., 2005) or when assessing the short‐term kinetic response as described here in our current study; however, our results do support a role for ARR2 in the hypocotyl based on the ethylene growth recovery kinetics of *arr2*. Differences observed between the two studies on *arr2* mutant phenotypes may relate to growth conditions used for studying the effects of ethylene on hypocotyl growth. In addition to potentially mediating ethylene effects on hypocotyl growth, ARR2 as well as three AHPs (AHP1, AHP2, and AHP3) have been implicated in mediating the stimulatory effects of ethylene on stomatal closure (Desikan et al., 2006; Mira‐Rodado et al., 2012). Furthermore, the type‐B response regulator ARR1, but not ARR2, ARR10, or ARR12, contributes to ethylene‐mediated inhibition of cell proliferation at the primary root meristem; kinase‐inactive versions of ETR1 similarly reduce this ethylene response, consistent with it being mediated through a phosphorelay initiated at the ethylene receptors (Street et al., 2015).

Our study supports the existence of two independent ethylene signaling pathways initiated at the receptors: the well‐documented CTR1/EIN2/EIN3‐dependent pathway as well as an AHP/ARR‐dependent pathway (Figure 5). The degree to which these pathways contribute to various ethylene responses varies depends on the response assayed. For example, both pathways contribute to a similar extent in ethylene‐mediated regulation of stomatal closure, but the CTR1/EIN2/EIN3 pathway is predominant in the regulation of cell proliferation at the root meristem (Desikan et al., 2006; Mira‐Rodado et al., 2012; Street et al., 2015). Although genetic analysis reveals a role for the AHP/ARR pathway in mediating the growth recovery response to ethylene, whether the CTR1/EIN2/EIN3 pathway also plays a role cannot be readily assessed because this pathway mediates the growth inhibition response (i.e., a growth recovery response cannot be assessed unless there is an initial growth inhibition response) (Binder, Mortimore, et al., 2004). As noted above, both pathways contribute to varying extents in the control of different ethylene responses. A potential mechanism that would facilitate such control is combinatorial regulation between the EIN3/EIL and type‐B ARR transcription factors, EIN3 and ARR having each individually been found to coregulate gene expression in conjunction with other transcription factors (Feng et al., 2017; Liu et al., 2017; Marin‐de la Rosa et al., 2015).

Through analysis of the hypersensitive response to ethylene, our results also indicate that the histidine kinase activity of ETR1 may play a transcriptionally independent role in ethylene signaling. Previous genetic analysis demonstrated that the hypersensitive response of Arabidopsis to ethylene is independent of the transcription factors EIN3 and EIL1 (Binder, Mortimore, et al., 2004), our results here demonstrating that this response is also independent of the type‐B ARR transcription factors ARR2, ARR10, and ARR12. Nevertheless, histidine kinase activity of ETR1 appears to play a role in the hypersensitive response, because loss of ETR1 or transformation of Arabidopsis with a kinase‐inactivated ETR1 results in a prolonged response. Because the ethylene receptors are components within larger signaling complexes (Chen et al., 2010), phosphorylation could potentially modulate the interactions and activity of proteins physically associated with ETR1 such as CTR1, EIN2, and/or other ethylene receptors (Clark et al., 1998; Gao et al., 2003, 2008; Grefen et al., 2008). However, the output in such a case would not be through an established transcriptionally based pathway but potentially through a phosphorylation/dephosphorylation cascade, which often mediates rapid eukaryotic signaling responses.

## AUTHOR CONTRIBUTIONS

BMB, JJK, and GES conceived and supervised the study. BMB and HJK designed and performed experiments. DEM and CEH generated mutant lines used in the study. BMB, HJK, and GES analyzed data. GES wrote the manuscript with contributions from all other authors.
