# Supplementary material for: A role for two‐component signaling elements in the Arabidopsis growth recovery response to ethylene
Source: Plant Direct. 2018 May 9;2(5):e00058. doi: 10.1002/pld3.58 (PMC6508545; doi:10.1002/pld3.58)
Supplement: Supplementary file 1 [file PLD3-2-e00058-s001.pdf]

Response to reviewers:

In the revised manuscript we have added the information requested by the reviewers, including (1) clarifying the effects of lovastatin on the seedlings; (2) adding a conclusion to the Abstract; (3) adding a summary/interpretation to the Introduction; and (4) making additional edits as recommended. These textual revisions are highlighted in yellow in the file 'Revised Manuscript with Highlights.' Specific responses to the reviewers are given below.

Sincerely,
G. Eric Schaller

<u>Reviewer #1:</u> The authors present a genetics-based study of some of the more understudied aspects of ethylene response and signal transduction. The paper is written clearly and the data are conclusive and support the major claims of the authors. I have no major concerns as the data and presentation are solid and supportive of the major conclusions.

No revisions were requested.

<u>Reviewer #2:</u> This manuscript focuses on testing the hypothesis that histidine kinase activity of the ethylene receptor ETR1 and activity of two-component system elements play a role in restoring normal seedling growth rates after ethylene treatment. In general, the manuscript is clearly written and the experiments include appropriate controls.

Data presented in the manuscript build on the prior observation that histidine kinase activity of ETR1 is required in a transgene complementation assay to restore normal growth recovery kinetics after ethylene treatment of an etr1 ers2 double loss-of-function mutant. Although AHKs 2, 3 and 4 are not required for growth rate recovery, AHPs 2, 3 and 5 as well as ARRs 2, 10 and 12 do play roles in restoring normal growth rates after ethylene treatment. Chemical inhibition of cytokinin biosynthesis does not mimic the effects of the ahp and arr mutations in the growth rate recovery assay, suggesting that these effects are independent of cytokinin response per se. Finally, ETR1 histidine kinase activity also contributes to the rapid recovery of growth rate during continuous, extremely low-dose ethylene treatment, while ARRs 2, 10 and 12 play little or no role in this response.

Although growth rate recovery occurs after the removal of the ethylene signal in the first set of experiments, and in the presence of ethylene in the last set, the time required for recovery is approximately 2 hours in both cases. This observation might have suggested a similar molecular basis for recovery in both assays; thus the demonstration of arr dependence in the former but not the latter assay reveals an important mechanistic distinction.

We thank the reviewer for their careful reading of the manuscript and thoughtful consideration of its main conclusions.

1. The authors should present data showing that lovastatin treatment used in Figure 3 does affect a normal cytokinin-dependent phenotype in treated plants.

In response to the reviewer's recommendation, we have provided additional information on lovastatin and its effects on the seedlings. The additional text in the Results section is as follows: "Lovastatin is an inhibitor of the cytosolic pathway for isoprenoid biosynthesis, one of the two pathways that generate the isopentenyl groups used in the biosynthesis of cytokinins. Treatment of four-day-old dark-grown seedlings with 1 µM lovastatin inhibited hypocotyl growth, reducing hypocotyl length from 9.87 mm (±0.15 SE) for the untreated control to 7.10 mm (±0.07 SE) for the lovastatin-treated seedlings (i.e. a 28% reduction in growth). When examined by kinetic analysis, the control seedlings exhibiting a growth rate of 0.33 mm/hr (± 0.01 SE) compared to 0.24 mm/hr (± 0.01 SE) for the lovastatin-treated seedlings (i.e. a 23% reduction in growth rate). This lovastatin-induced reduction in hypocotyl growth is consistent with that previously observed (Nagata et al., 2002), is indicative of the efficacy of the inhibitor, and also still allowed sufficient growth to perform kinetic analysis (Fig. 3)."

2. The writing could be improved in a few places in the manuscript. The abstract lacks any statement about the authors' conclusions, and the end of the introduction does not provide a summary of the data or its interpretation.

In response to the reviewer's recommendation, we have revised the abstract, incorporating this additional text: "The ability of two-component signaling elements to regulate the growth recovery response to ethylene functions independently from their well-characterized role in cytokinin signaling, based on the analysis of cytokinin receptor mutants as well as following chemical inhibition of cytokinin biosynthesis. Histidine-kinase activity of the receptor ETR1 also facilitates growth recovery in the ethylene hypersensitive response, which is characterized by a transient decrease in growth rate when seedlings are treated continuously with a low dose of ethylene; however, this response was found to operate independently of the type-B response regulators. These results indicate that histidine-kinase activity of the ethylene receptor ETR1 performs two independent functions: (1) regulating the growth recovery to ethylene through a two-component signaling system involving phosphotransfer proteins and type-B response regulators, and (2) regulating the hypersensitive response to ethylene in a type-B response regulator independent manner."

We have also revised the end of the introduction to incorporate this additional text: "Results from this study indicate that histidine-kinase activity of the ethylene receptor ETR1 performs two independent functions. First, it regulates the growth recovery to ethylene through a two-component signaling system involving AHPs and type-B ARRs. Second, it also plays a role in regulating growth recovery during a "hypersensitive" response to ethylene, which is characterized by a transient decrease in growth rate when seedlings are treated with a continuous very low dose of ethylene, but does so in a type-B

ARR independent manner. The potential mechanisms underlying these differences in histidine-kinase mediated regulation are discussed."

3. Some additional specific suggestions are detailed below.

Line 36 "these" is confusing - refers to histidine kinases? (delete)
Line 90 ...independently of CTR1, supporting the possibility of other,...
Line 162 The two Binder 2004 references need discriminators.
Line 162-3 ...the growth response has two kinetic phases
Line 167-8 ... affect these growth recovery kinetics (Binder...
Line 208 ...confirm the lack of correlation between the effects of the...
Line 212 ...were more resistant to cytokinin OR showed less response to cytokinin ("more hyposensitive" is quite awkward)
Line 217-8 ...are not affected by lovastatin, ...
Line 287 ... similarly reduce this ethylene response ...

We have made these changes as recommended to clarify the manuscript.
